# Increasing the sensitivity of hyperpolarized [^15^N_2_]urea detection by serial transfer of polarization to spin‐coupled protons

**DOI:** 10.1002/mrm.28241

**Published:** 2020-03-10

**Authors:** Felix Kreis, Alan J. Wright, Vencel Somai, Rachel Katz‐Brull, Kevin M. Brindle

**Affiliations:** ^1^ Cancer Research UK Cambridge Institute University of Cambridge Cambridge UK; ^2^ Department of Radiology Faculty of Medicine Hadassah Medical Center Hebrew University of Jerusalem Jerusalem Israel; ^3^ Department of Biochemistry University of Cambridge Cambridge UK

**Keywords:** hyperpolarization, indirect detection, nitrogen‐15, urea

## Abstract

**Purpose:**

Hyperpolarized ^15^N‐labeled molecules have been proposed as imaging agents for investigating tissue perfusion and pH. However, the sensitivity of direct ^15^N detection is limited by the isotope's low gyromagnetic ratio. Sensitivity can be increased by transferring ^15^N hyperpolarization to spin‐coupled protons provided that there is not significant polarization loss during transfer. However, complete polarization transfer would limit the temporal window for imaging to the order of the proton T_1_ (2‐3 s). To exploit the long T_1_ offered by storing polarization in ^15^N and the higher sensitivity of ^1^H detection, we have developed a pulse sequence for partial polarization transfer.

**Methods:**

A polarization transfer pulse sequence was modified to allow partial polarization transfer, as is required for dynamic measurements, and that can be implemented with inhomogeneous B_1_ fields, as is often the case in vivo. The sequence was demonstrated with dynamic spectroscopy and imaging measurements with [^15^N_2_]urea.

**Results:**

When compared to direct ^15^N detection, the sequence increased the signal‐to‐noise ratio (SNR) by a factor of 1.72 ± 0.25, where both experiments depleted ~20% of the hyperpolarization (>10‐fold when 100% of the hyperpolarization is used). Simulations with measured cross relaxation rates showed that this sequence gave up to a 50‐fold increase in urea proton polarization when compared to spontaneous polarization transfer via cross relaxation.

**Conclusion:**

The sequence gave an SNR increase that was close to the theoretical limit and can give a significant SNR benefit when compared to direct ^13^C detection of hyperpolarized [^13^C]urea.

## 1 INTRODUCTION

Magnetic resonance imaging of hyperpolarized isotopically labeled substrates has enabled measurements of metabolic fluxes, pH, and tissue perfusion in vivo. The most commonly used label has been ^13^C because of its relatively long polarization lifetime and the availability of ^13^C‐labeled substrates suitable for investigating metabolism.^1^ Hyperpolarized ^15^N‐labeled substrates have also been investigated, as agents for assessing tissue perfusion (urea^2^ and glutamine^3^) and as pH probes (pyridine derivatives).^4^ ^15^N labeled substrates have the advantage of very long hyperpolarization lifetimes, up to 200 s, and more when kept in ^2^H_2_O.^2^ However, the 2.5‐fold lower gyromagnetic ratio when compared to ^13^C (10‐fold lower when compared to ^1^H) results in lower magnetization and precession frequency and therefore lower sensitivity of detection. For imaging there is also the requirement for larger gradients.

Detection sensitivity can be improved, while still benefiting from the long ^15^N polarization lifetime, using sequences such as insensitive nuclei enhanced by polarization transfer (INEPT) to transfer hyperpolarization from ^15^N to ^1^H immediately before signal acquisition.^5^ Reverse INEPT‐type sequences have been used previously with hyperpolarized ^13^C‐labeled substrates to produce hyperpolarized proton spectra^6^, ^7^, ^8^, ^9^ and images^10^, ^11^ and with hyperpolarized ^15^N labeled substrates to produce spectra.^12^, ^13^, ^14^ In all of these INEPT‐based experiments 100% of the available polarization was used in a single acquisition. To obtain dynamic information the hyperpolarization of the low γ nucleus must be sampled in discrete packets in order to allow repeat measurements, which in the case of direct ^15^N or ^13^C detection is achieved using small flip angle (FA) pulses. For example, in the case of dynamic perfusion measurements with hyperpolarized [^15^N_2_]urea only a portion of the ^15^N polarization should be transferred to the urea protons at each measurement. The same is true for measurements of flux in an enzyme‐catalyzed reaction, for example exchange of hyperpolarized ^13^C label between injected [1‐^13^C]pyruvate and the endogenous lactate pool.

**FIGURE 1:**
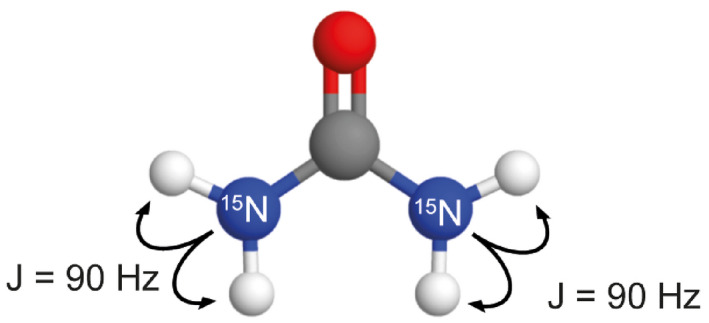
Structure of [^15^N_2_]urea. The coupling constant between ^15^N and the directly bonded protons is 90 Hz. These protons are in exchange with solvent water

Several approaches have been taken to achieve partial transfer of polarization. Harris et al^13^ used spatially selective coherence transfer to probe different regions of the sample at different times. Barb et al exploited chemical exchange of deuterons in hyperpolarized ^15^ND_2_‐amido‐glutamine with solvent protons to acquire a series of proton spectra from the protonated isotopologue.^12^ Dzien et al^7^ utilized spontaneous ^13^C → ^1^H cross‐relaxation to detect, in a series of dynamically acquired proton spectra, the production of acetaldehyde from hyperpolarized [U‐^2^H_3_,2‐^13^C]pyruvic acid, in the reaction catalyzed by pyruvate decarboxylase. We have previously described a spectrally selective reverse INEPT sequence in which ^13^C hyperpolarization in lactate, which had been produced in a tumor from injected hyperpolarized [1‐^13^C]pyruvate, was transferred to the methyl protons and imaged.^11^ In this case the fully depleted [1‐^13^C]lactate hyperpolarization was replenished after each transfer by further labeled lactate production from the injected pyruvate. However, to the best of our knowledge, no one has yet demonstrated experimentally partial transfer of hyperpolarization from a low γ to a high γ nucleus, while maintaining the majority of the hyperpolarization in the low γ nucleus. We demonstrate here partial transfer of ^15^N hyperpolarization in [^15^N_2_]urea to urea protons (Figure 1) in consecutive acquisitions and subsequent imaging of these protons, as would be required for dynamic imaging of tissue perfusion. The sensitivity of urea proton detection in this experiment was compared with direct ^15^N detection and, in simulations using measured cross relaxation rates, with proton detection where polarization is transferred spontaneously from ^15^N to ^1^H via the nuclear Overhauser enhancement (NOE).

## 2 METHODS

### 2.1 Solvent exchange of [^15^N_2_]urea protons

The exchange rate in a 100 mM [^15^N_2_]urea solution was measured at neutral pH using a 14.1 T nuclear magnetic resonance (NMR) spectrometer equipped with a 5 mm BBI probe (Bruker Spectrospin Ltd., Coventry, UK). The water proton resonance was saturated for between 0.1 and 2.6 s and then spectra acquired using a 90° pulse and a bandwidth of 6000 Hz into 8192 complex points. The exchange rate was calculated as described in Ref. 15.

### 2.2 Relaxation times

^1^H and ^15^N relaxation times in [^15^N_2_]urea were measured on an Agilent 7 T preclinical scanner (Agilent, Palo Alto, California) using a home‐built single turn dual‐tuned ^1^H/^15^N transmit/receive surface coil^16^ with an inner diameter of 14 mm. The same coil was used for polarization transfer experiments. The sample contained 3 mL of 1 M [^15^N_2_]urea in phosphate‐buffered saline (PBS) and 300 μL ^2^H_2_O at 20°C. *T*_1_ relaxation times were measured with an inversion recovery sequence (n* = *1, *TR*_1*H*_ = 10 s, *TR*_15*N*_ = 100 s). The time between the 90° and 180° pulses was varied between 0.25 and 8 s for the ^1^H measurements and between 2.5 and 80 s for the ^15^N measurements. *T*_2_ relaxation times were measured with a Carr‐Purcell‐Meiboom‐Gill (CPMG) sequence (n* = *1, *TR*_1*H*_ = 10 s, *TR*_15*N*_ = 100 s). The minimum echo time for the ^1^H measurements was 0.0125 s, which was increased by iteratively adding more spin echo sandwiches while leaving the inter echo spacing the same until, over six acquisitions, the maximum echo time of 0.4 s was reached. For ^15^N *T*_2_ measurements the echo time was varied between 0.0624 and 2 s.

### 2.3 Dynamic nuclear polarization

Samples were prepared from 45.9 mg [^15^N_2_]urea, 2.31 mg OXO63 trityl radical, 62.8 mg ^2^H_2_O, and 55.4 mg glycerol. The mixture (37.5 mg) was polarized for at least 3 hours in a *HyperSense* polarizer (Oxford Instruments, Abingdon, UK) using microwave irradiation at 94.110 GHz. Dissolution was performed in 6 mL ^2^H_2_O because this has been shown to prolong the ^15^N T_1_ (2). [^13^C]urea was hyperpolarized as described by von Morze et al.^17^ The [^13^C]urea preparation contained 6.4 M [^13^C]urea and 23 mM OXO63 in glycerol. The preparation (29.4 mg) was polarized at 94.095 GHz and dissolved in 6 mL PBS.

### 2.4 Polarization measurements

Spectra were acquired using a 90° pulse and a sweep width of 20 kHz into 16 384 complex points from 4 mL of hyperpolarized urea using a 14.1 T NMR spectrometer and a 10 mm BBO probe (Bruker Spectrospin Ltd.) at room temperature. The signal‐to‐noise ratio (SNR) was compared to that in spectra of the same solution after decay of the hyperpolarization. For these experiments, measurements at thermal equilibrium from [^13^C]urea were acquired with a pulse repetition time (TR) of 225 s and were the sum of 237 averages. For [^15^N_2_]urea the TR was 1000 s and 32 averages were acquired. The thermal polarization:

(1) $$\begin{array}{c} P_{\mathrm{thermal}}=\tanh\left(\frac{\hbar \gamma B_{0}}{2k_{B}T}\right) \end{array}$$

was calculated to be 4.87 × 10^−6^ for ^15^N and 12.08 × 10^−6^ for ^13^C, assuming a temperature of 300 K, where ℏ is the reduced Planck's constant, *ɣ* the gyromagnetic ratio, *k_B_* Boltzmann's constant, and *B*_0_ = 14.1 T. The hyperpolarization, *P_hyp_*, was calculated from the SNRs of the hyperpolarized and thermal measurements using:

(2) $$\begin{array}{c} P_{\mathrm{hyp}}=P_{\mathrm{thermal}}\sqrt{n_{\mathrm{avg}}}\;\frac{SNR_{\mathrm{hyp}}}{SNR_{\mathrm{thermal}}} \end{array}$$

The measured polarization was 6.2% for [^13^C]urea and 2.3% for [^15^N_2_]urea. The value for ^15^N was lower than the 5% reported previously,^2^ and the value for ^13^C was between a value of 3% reported in vivo^17^ and a value of 10% estimated at the time of injection.^18^

### 2.5 Measurement of coil performance

A cylindrical phantom containing 2 mL of 4 M [^15^N_2_]urea was placed through the loop of the dual‐tuned resonator and ^1^H and ^15^N spectra acquired with a sweep width of 10 kHz into 2048 complex points with one average using a 2 ms BIR4 90° pulse, with pulse shape parameters as described by Merkle et al.^19^ Coil performance at the ^1^H and ^15^N frequencies was assessed by comparing the SNRs of the two spectra.

### 2.6 Pulse sequence

The INEPT pulse sequence,^5^ which transfers polarization from S to I spins via J‐coupling, can be written as:

$$S:\;90_{x}^{\circ }\;-\;\tau_{1}\;-\;180_{x}^{\circ }\;-\;\tau_{2}\;-\;90_{y}^{\circ }$$

$$I:\;180_{x}^{\circ }\;-\;\tau_{2}\;-\;90_{x}^{\circ }\;-\;\;\text{Acq}.$$

where full transfer of polarization is achieved when *τ*_1,2_ = 1/(4 *J*). Simultaneous application of 90° pulses to the I and S spins converts an antiphase state of the S spins to an observable antiphase state in the I spins. A later version of this sequence^20^ refocuses the I spin magnetization.

$$S:\;90_{y}^{\circ }\;-\;\tau_{1}\;-\;180_{x}^{\circ }\;-\;\tau_{2}\;-\;90_{x}^{\circ }\;-\;\tau_{3}-180_{x}^{\circ }\;-\;\tau_{4}\;-\;\;\text{Dec}.$$

$$I:\;90_{y}^{\circ }-\tau_{1}-180_{x}^{\circ }-\tau_{2}-90_{y}^{\circ }-\tau_{3}-180_{x}^{\circ }\;-\;\tau_{4}-\;\text{Acq}.$$

In an IS spin system full transfer occurs when all the delays *τ*_1,2,3,4_ are 1/(4* J*)*.* Merkle et al^19^ later described a sequence that used composite pulses to compensate for *B*_1_ inhomogeneity:

$$\begin{array}{c} S:\;\left[90_{y}^{\circ }-\tau_{1}-180_{\left(y+5\pi /4\right)}^{\circ }-\tau_{2}-90_{y}^{\circ }\right]-\tau_{3}-\left[90_{y}^{\circ }180_{\left(y+3\pi /2\right)}^{\circ }90_{y}^{\circ }\right]-\tau_{4}-\text{Dec}. \end{array}$$

$$I:\;\left[90_{y}^{\circ }-\tau_{1}-180_{\left(y+\pi \right)}^{\circ }-\tau_{2}-90_{y}^{\circ }\right]-\tau_{3}-\left[90_{y}^{\circ }180_{\left(y+3\pi /2\right)}^{\circ }90_{y}^{\circ }\right]-\tau_{4}-\text{Acq}.$$

These composite pulses were later replaced with modified BIR4 pulses to produce the BINEPT sequence, which is the basis of the pulse sequence described here.

**FIGURE 2:**
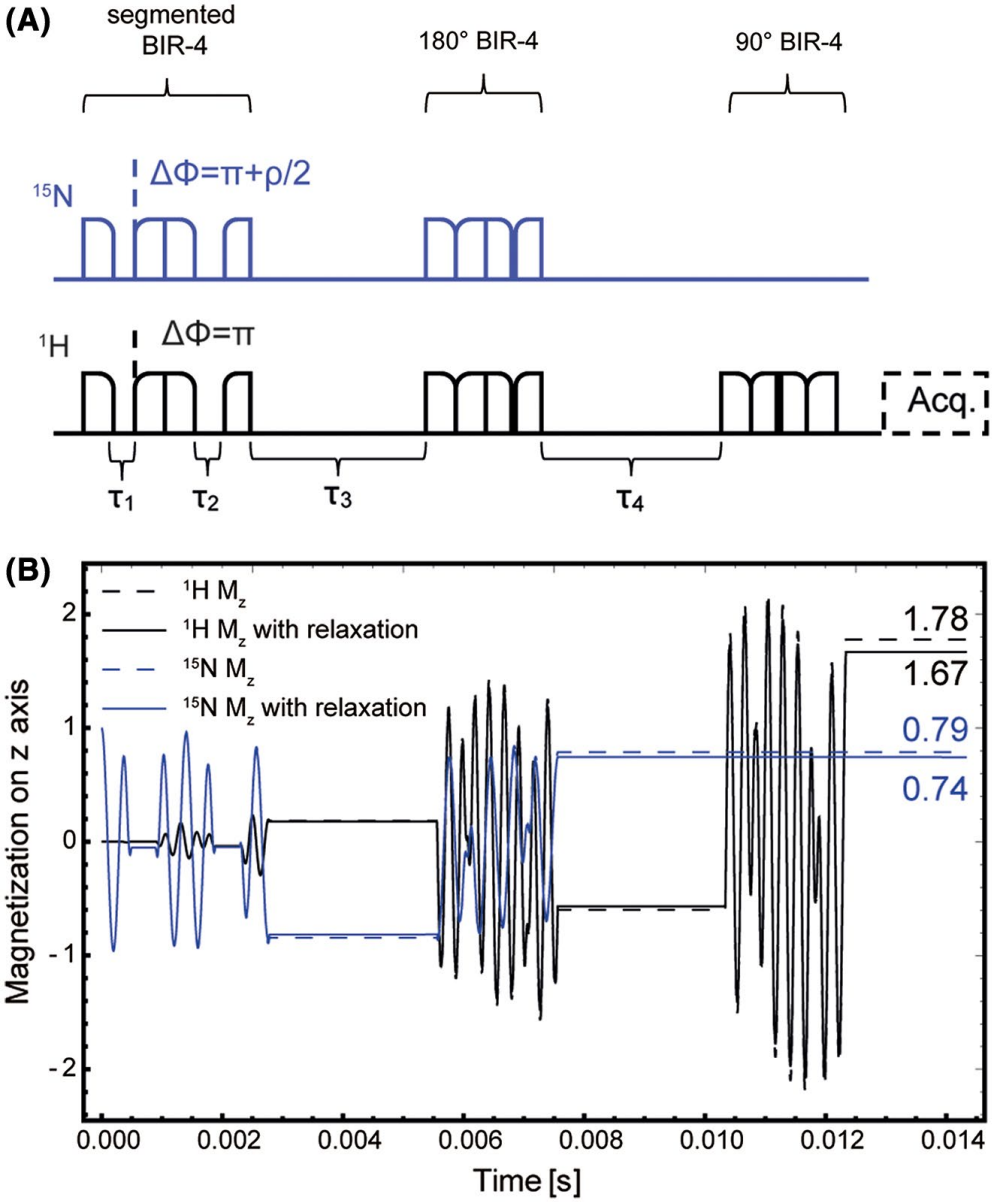
Timing (A) and simulation (B) of the ImpeRfection RobUst Partial Transfer (IRRUPT) polarization transfer pulse sequence, which is based on the B1‐insensitive nuclear enhancement throughpolarization transfer (BINEPT)^19^ and hyperpolarized insensitive nucleus delivers enhancement repeatedly (HINDER)^22^ sequences, with *τ*_1_ + *τ*_2_ = 0.442/(2 *π J_NH_*) = 782 µs, *τ*_3_ + *τ*_4_ = 1/(2* J_NH_*)* = *5555 µs, *δ = *18.050°. The sequence was simulated with a maximum B_1_ amplitude of 2500 Hz for ^15^N and 4000 Hz for ^1^H

A BIR4 pulse^21^ is composed of three sections: adiabatic half‐passage in reverse, adiabatic inversion, and adiabatic half‐passage. The FA is controlled by two phase jumps Δ*ϕ*_1_ and Δ*ϕ*_2_ = −Δ*ϕ*_1_ before and after the adiabatic inversion segment respectively. The transformation induced by a BIR4 pulse can be described using a composite pulse analogy, where ($90y^{\circ }180^{\circ }\left(y+\pi +\delta /2\right)90y^{\circ }\;$) is analogous to a BIR4 pulse with phase jumps Δ*ϕ*_1_ = −Δ*ϕ*_2_ = *π+δ*/2. Both the BIR4 pulse and this composite pulse execute a rotation of *δ* rad about the *x* axis, although a composite pulse requires a considerably more uniform *B*_1_ field to achieve this transformation. With this simplification, the BINEPT sequence can be written as:

$$S:\;\left[90_{y}^{\circ }-\tau_{1}-180_{\left(y+\pi +\delta /2\right)}^{\circ }-\tau_{2}-90_{y}^{\circ }\right]-\tau_{3}-\left[90_{y}^{\circ }180_{\left(y+3\pi /2\right)}^{\circ }90_{y}^{\circ }\right]-\tau_{4}-$$

$$I:\;\left[90_{y}^{\circ }-\tau_{1}-180_{\left(y+\pi \right)}^{\circ }-\tau_{2}-90_{y}^{\circ }\right]-\tau_{3}-\left[90_{y}^{\circ }180_{\left(y+3\pi /2\right)}^{\circ }90_{y}^{\circ }\right]-\tau_{4}-\;\text{Acq}.$$

where for this example the I spin is proton and the S spin ^15^N. For *δ = π*/2 this is identical with the composite pulse sequence and is analogous to the BINEPT sequence using adapted BIR4 pulses in terms of net rotations; however, the paths taken by the magnetization vectors differ. In this simplified sequence, the phase offset *δ* of the first 180° pulse on the S spin must be 90° and *τ*_1‐4_ must be 1/(4 *J*) in order to fully transfer polarization from the S to the I spin in a two‐spin system. When *τ_1_* and *τ_2_* are shortened and the phase offset *δ* adjusted, some of the magnetization can be returned to the *z* axis while still transferring some of the polarization. For a simple IS spin system the transferred polarization (*P_I_*) is equal to sin*(δ)*sin*(π J τ)P*_0_ and the polarization returned to the *z* axis (*P_S_)* is equal to −cos*(δ)*cos*(π J τ)P*_0_, where *P*_0_ is the original polarization, *J* is the coupling constant between the I and S spins and *τ* = 2τ_1_ = 2*τ*_2_ (see product operator analysis in the Supporting Information Text S1). In all cases, *τ*_3_ =* τ*_4_ = 1/(4 *J*). For an *IS_N_* spin system these terms are *P_I_ = N* sin*(δ)*sin*(π J τ)* and *P_S_ = *−cos*(δ)[*cos*(π J τ)] ^N^*. A similar approach has been described previously in the HINDER sequence (*h*yperpolarized *i*nsensitive *n*ucleus *d*elivers *e*nhancement *r*epeatedly),^22^ where spin order is divided between I or S spin polarization by changing the phase, *δ*, of the second 90° pulse on the S spin and shortening the inter pulse delays in the classical INEPT sequence. To summarize, we have combined the BINEPT and HINDER sequences to give an ImpeRfection RobUst Partial Transfer (IRRUPT) sequence, where *δ* and *τ*_1_ and *τ*_2_ in the BINEPT sequence can be adjusted to achieve partial polarization transfer. An additional 180 degrees was added to *δ* to return the remaining magnetization to the positive instead of the negative axis (making *P_I_ = *−cos*(δ)[*cos*(π J τ)] ^N^* positive). The delays between the pulses (*τ*_1‐4_) and the additional phase offset *δ* in the segmented BIR4 pulse on the S spin were chosen as described for the HINDER sequence.^22^ For two protons coupled with a 90 Hz coupling constant (*J*) to one low γ nucleus: *τ*_1_ + *τ*_2_ = 0.442/(2* π J) = *782* *µs, *τ*_3_ + *τ*_4_ = 1/(2* J) = *5555* *µs, *δ = *18.050°. The delays were not corrected for the fact that relatively long adiabatic pulses were used instead of hard pulses. With adiabatic pulses in the simulations these parameters resulted in ~20% of the ^15^N hyperpolarization being transferred to the coupled protons (*J*_*NH*_ = −90 Hz) (Figure 2). Each adiabatic half passage segment in the BIR4 pulse was 500 μs, giving a total pulse duration of 2 ms The last BIR4 pulse in the sequence flips the proton magnetization onto the *z* axis. Then, either a simple excitation pulse or a slice‐selective 2D‐single‐shot echo‐planar imaging (EPI) sequence was used for proton acquisition. In both cases a 90° pulse was used in order to make maximum use of the transferred polarization.

### 2.7 Simulation of the IRRUPT pulse sequence

**FIGURE 3:**
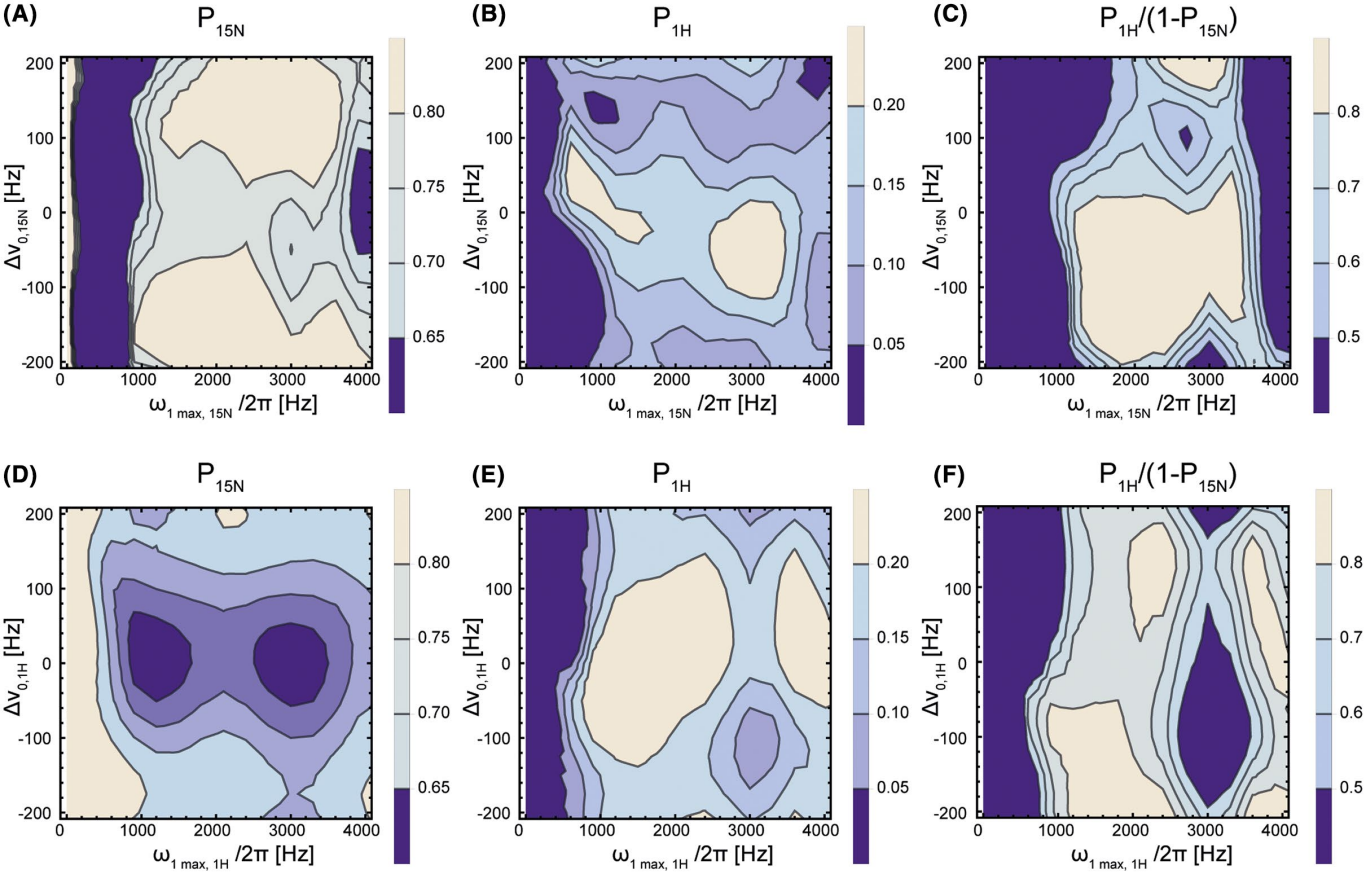
Simulations of the ImpeRfection RobUst Partial Transfer (IRRUPT) pulse sequence. Remaining ^15^N polarization (P_15N_) after one transfer as a function of (A) ^15^N excitation frequency offset and ^15^N maximum pulse amplitude and (D) ^1^H excitation frequency offset and ^1^H maximum pulse amplitude. ^1^H polarization (P_1H_) after one transfer as a function of (B) ^15^N excitation frequency offset and ^15^N maximum pulse amplitude and (E) ^1^H excitation frequency offset and ^1^H maximum pulse amplitude. Polarization transfer efficiency P_1H_/(1−P_15N_) as a function of (C) ^15^N excitation frequency offset and ^15^N maximum pulse amplitude and (F) ^1^H excitation frequency offset and ^1^H maximum pulse amplitude. The asymmetry in the profiles results from the use of adiabatic pulses

Evolution of the ^15^N and ^1^H magnetizations were simulated using *SpinDynamica* ^23^ (www.spindynamica.soton.ac.uk) in Wolfram Mathematica (version 11; Wolfram Research, Inc, Champaign, Illinois). The Hamiltonian describing the [^15^N_2_]urea spin system is:

(3) $$\begin{array}{c} {\hat{H}}_{0}=2\pi J_{\mathrm{NH}}\left({\hat{S}}_{z}{\hat{I}}_{z,1}+\;{\hat{S}}_{z}{\hat{I}}_{z,2}\right)\; \end{array}$$

where *J_NH_* is the negative heteronuclear coupling constant,^24^ $\hat{S}$ is the product operator for the ^15^N spin, and ${\hat{I}}_{1}$ and ${\hat{I}}_{2}$ the operators for the two equivalent protons. To simulate the effect of off‐resonance excitation, the term

(4) $$\begin{array}{c} {\hat{H}}_{\text{off}}\;=2\pi v_{0,1H}\left({\hat{I}}_{z,1}+{\hat{I}}_{z,2}\right)+2\pi v_{0,15N}{\hat{S}}_{z} \end{array}$$

was added to the ${\hat{H}}_{0}$ Hamiltonian. *v_0,15N_* and *v_0,1H_* are the excitation frequency offsets in Hz. To estimate relaxation losses the *PhenomenologicalRelaxationSuperoperator* function in *SpinDynamica* was used with the measured *T*_1_ and *T*_2_ times. An uncorrelated relaxation model was assumed. We define the proton polarization P_1H_ divided by the depleted ^15^N polarization (1‐*P*_15*N*_) at the end of the transfer block as the efficiency of polarization transfer (*efficiency* = *P*_1*H*_/(1−*P*_15*N*_)), which was typically >80% (Figure 3).

### 2.8 Polarization transfer in phantom experiments

Polarization transfer experiments were performed on the 7 T scanner using the dual‐tuned home‐built surface coil. A spherical phantom filled with 3 mL water was positioned in the magnet isocenter. For hyperpolarized acquisitions, 1.5 mL of water were removed and replaced with 1.5 mL hyperpolarized [^15^N_2_]urea solution. Spectra were acquired with a 2 ms 90° BIR4 excitation pulse after the polarization transfer block, with TR = 2s, sweep width = 100 kHz, number of points = 4096. Images were acquired with a 2D EPI sequence after the polarization transfer block. Six water presaturation pulses with crusher gradients were used prior to the polarization transfer block for both imaging and spectroscopy experiments. Images were acquired with FOV* = *32 × 32 × 1 mm, TR* = *1s, bandwidth = 250 kHz, matrix size = 32 × 32.

### 2.9 Interleaved direct and indirect detection

The SNR benefits of indirect over direct detection were determined by interleaving the two acquisition strategies. After injection of 1.5 mL hyperpolarized [^15^N_2_]urea three direct detection ^15^N spectra were acquired using 30° pulses followed by three indirect polarization transfer measurements (with no water pre saturation), then three direct detection measurements followed by another three indirect detection measurements. Each spectrum was phase and baseline corrected and then normalized to the standard deviation of the noise in the first 400 points at the downfield end of the spectrum. The indirect detection ^1^H spectra were further corrected to account for depletion of polarization due to the prior direct ^15^N detection. This was achieved by multiplying spectra 4, 5, and 6 (indirect detection) by three times the reciprocal of the average signal loss between spectra 1 and 2 and between spectra 2 and 3 (direct detection). Spectra 10, 11, and 12 were corrected in a similar way but using a factor calculated from the signal loss between spectra 7 and 8 and between spectra 8 and 9. The ^1^H spectra were recorded with a sweep width of 10 kHz into 2048 complex points, an acquisition time of 204.8 ms and a TR of 500 ms The directly detected ^15^N spectra were acquired with a nominal 30° FA pulse, sweep width 10 kHz, 2048 complex points, TR = 500 ms, which were the same acquisition parameters as used for the indirect measurements. The SNRs were calculated by integrating a 60 point‐wide region containing the peak and dividing it by the standard deviation of the noise in the 400 point‐wide region at the downfield end of the spectrum.

### 2.10 Simulations of spontaneous polarization transfer from ^15^N to ^1^H via cross relaxation

**FIGURE 4:**
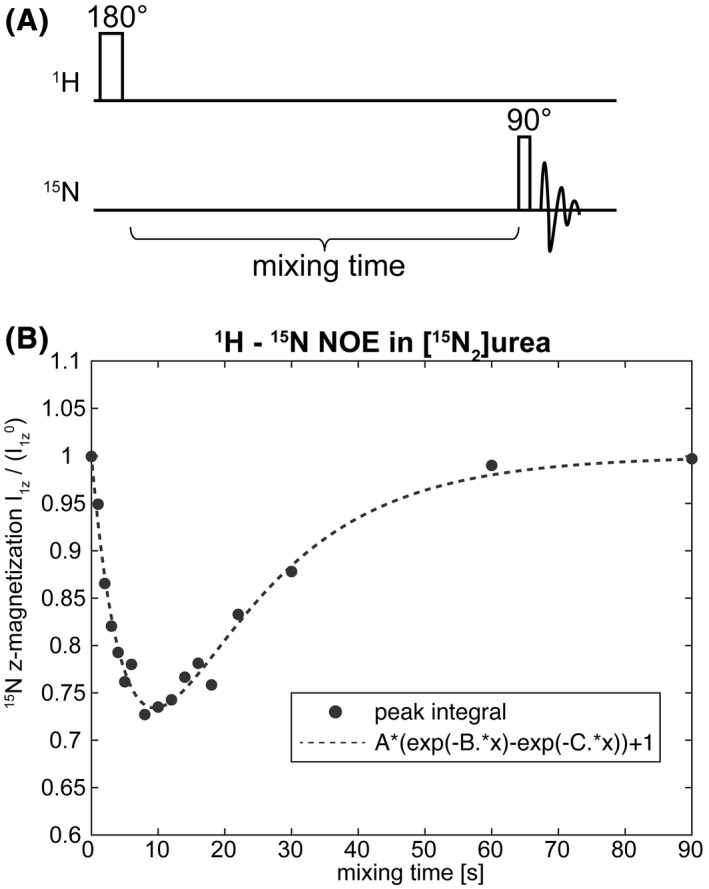
A, Pulse sequence used to measure the ^1^H−^15^N cross relaxation rate in a 500 mM [^15^N_2_]urea sample. B, Plot of ^15^N signal versus mixing time. Signal intensity was fitted to an analytical solution of the Solomon equations (Equations 5 and 6). NOE, nuclear Overhauser enhancement

Heteronuclear cross relaxation rates in [^15^N_2_]urea were measured by inverting the water and urea ^1^H resonances in a 500 mM thermally polarized [^15^N_2_]urea solution and observing the ^15^N resonance (Figure 4). Measurements were made at 310 K using a 5 mm Bruker BBI probe at 14.1 T, with 10% ^2^H_2_O for a field‐frequency lock. The ^1^H and ^15^N magnetizations can be described by the following equations:

(5) $$\begin{array}{c} \frac{dI_{1z}\left(t\right)}{\mathrm{dt}}=-R_{z}^{\left(1\right)}\left(I_{1z}\left(t\right)-I_{1z}^{0}\right)-\sigma_{12}\left(I_{2z}\left(t\right)-I_{2z}^{0}\right)\; \end{array}$$

(6) $$\begin{array}{c} \frac{dI_{2z}\left(t\right)}{\mathrm{dt}}=-\sigma_{21}\left(I_{1z}\left(t\right)-I_{1z}^{0}\right)-R_{z}^{\left(2\right)}\left(I_{2z}\left(t\right)-I_{2z}^{0}\right). \end{array}$$

Where *I*_1_ is the ^15^N and *I*_2_ the ^1^H magnetizations. Following the 180° ^1^H pulse at *t* = 0:

(7) $$\begin{array}{c} I_{1z}\left(0\right)\;=\;I_{1z}^{0} \end{array}$$

(8) $$\begin{array}{c} I_{2z}\left(0\right)\;=\;-I_{2z}^{0} \end{array}$$

For this boundary condition the system of differential equations (Equations (5), (6)) has a solution of the form $f\left(t\right)\;=\;a\left(e^{-Bt}-e^{-Ct}\right)$, where, from an initial rate approximation, the cross relaxation rate between ^1^H and ^15^N ($\sigma_{12})=\frac{a\left(C-B\right)}{2}\frac{\gamma_{15N}}{\gamma_{1H}}$.^25^ This describes the combined cross relaxation between water (intermolecular NOE) and urea protons (intramolecular NOE) and the nitrogen‐15 in [^15^N_2_]urea and represents an upper limit for the intramolecular relaxation rate. From this an upper limit for the reverse rate (^15^N to ^1^H), $\sigma_{21}$, was calculated using the relation $\sigma_{21}=\frac{N_{1}}{N_{2}}\sigma_{12}$, where N_1_/N_2_ is the concentration ratio of the nuclei.^26^ We were interested in only intramolecular cross relaxation, because this would give the greatest enhancement, N_1_/N_2_ = 0.5. In the case of intermolecular cross relaxation the ^15^N hyperpolarization would be diluted among the many participating water protons and give a much smaller ^1^H enhancement.^26^ The measured cross relaxation rates $\sigma_{12}$ and $\sigma_{21}$ were then used to calculate the spontaneous transfer of polarization from ^15^N to urea protons that would occur in hyperpolarized [^15^N_2_]urea. The degree of transfer was compared with that produced by repeated application of the IRRUPT sequence, calculated using *SpinDynamica*.

## 3 RESULTS

Implementation of polarization transfer sequences using a surface coil requires the use of inversion pulses that compensate for B_1_ inhomogeneity. Furthermore, for dynamic measurements, polarization should be transferred in discrete packets from the hyperpolarized lower γ nucleus, in this case ^15^N, to the detected high γ nucleus, in this case ^1^H. We have modified the previously described BINEPT sequence,^19^ which uses BIR4 adiabatic pulses,^21^ for partial and sequential transfer of polarization from ^15^N to ^1^H (see Methods section for details).

### 3.1 Simulations of the IRRUPT pulse sequence

The sequence was simulated with the delays and phases used experimentally (*τ*_1_ + *τ*_2_ = 0.442/(2* π J_NH_*)* = *782* *µs, *τ*_3_ + *τ*_4_ = 1/(2* J_NH_*)* = *5555* *µs, *δ = *18.050°), which resulted in 21% of the ^15^N polarization being transferred to ^1^H and a ^1^H z‐magnetization that was 1.78 times greater than the initial ^15^N z‐magnetization (Figure 2B). Polarization loss due to relaxation is minimal because transfer via the strong coupling (J_NH_ = −90 Hz) is fast (~12 ms) and the simulation (Figure 2B) showed that this can be neglected. Simulations using measured relaxation rates (Table 1), showed that these values were reduced only slightly, from 79% to 74% for the remaining ^15^N magnetization and from 1.78 to 1.67 for the ^1^H magnetization. In further simulations the effect of the pulse sequence was simulated for a large range of excitation frequency offsets and pulse amplitudes (Figure 3). Transfer efficiency was preserved for large regions of parameter space, demonstrating the sequence's insensitivity to B_1_ inhomogeneity.

**TABLE 1:** ^1^H and ^15^N relaxation times in [^15^N_2_]urea measured at 7 T. 1 M [^15^N_2_]urea in phosphate‐buffered saline containing 10% ^2^H_2_O at 20°C

[^15^N_2_]urea	*T*_1_ relaxation time (s)	*T*_2_ relaxation time (s)
^1^H	2.57 ± 0.08	0.060 ± 0.003
^15^N	24.22 ± 1.15	1.62 ± 0.94

### 3.2 Effect of solvent exchange on urea proton hyperpolarization

The polarization transferred from ^15^N to the urea protons will be diluted by exchange with solvent water protons, decreasing the sensitivity of detection. However, this effect is small. The proton exchange rate between urea and water was determined by fitting the peak integrals following saturation *I*(*t_sat_*) to the equation given by Horska and Spencer^15^:

(9) $$\begin{array}{c} I\left(t_{\mathrm{sat}}\right)=c\left[1+kT_{1}e^{-\left(1/1T_{1}+k\right)t_{\mathrm{sat}}}\right] \end{array}\;$$

where *c* is a dimensionless factor, *T*_1_ is the urea proton relaxation time, *t_sat_* are the presaturation times, and *k* the exchange rate. This gave *c =* (1.14 ± 0.09), *k =* (1.56 ± 0.15) s^−1^, and *T*_1_ = (2.73 ± 0.38) s. The errors are those for the fitting. The measured lifetime for a proton in urea (1/*k*) was 0.64 ± 0.06 s, which is similar to that measured previously for 1 M urea at pH 7 (0.55 s).^27^ The IRRUPT sequence, including the flip‐back pulse, takes only ~12 ms and therefore the effect of solvent exchange on the urea proton hyperpolarization can be ignored.

### 3.3 Experimental implementation of the pulse sequence

**FIGURE 5:**
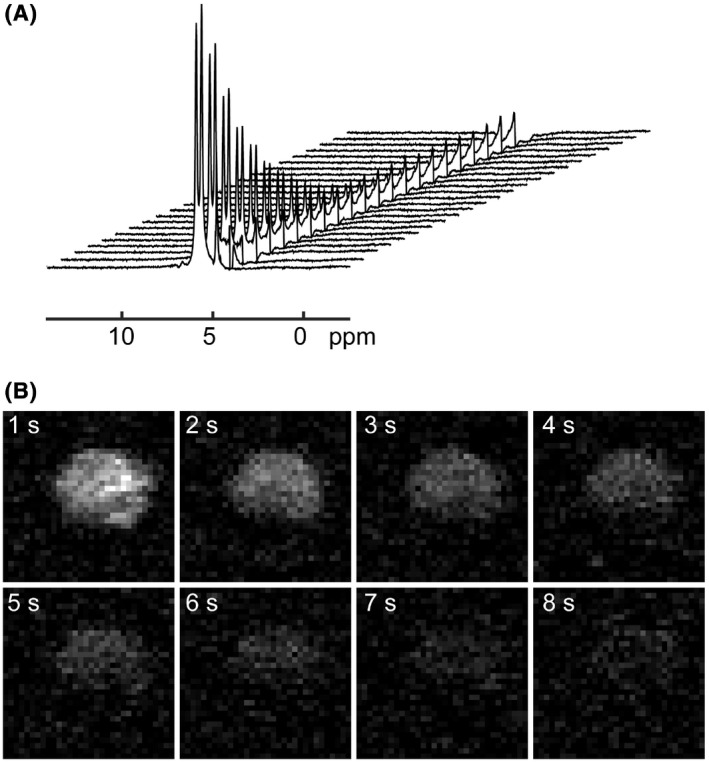
A, Dynamic ^1^H spectra of hyperpolarized [^15^N_2_]urea. Each spectrum was acquired with an ImpeRfection RobUst Partial Transfer (IRRUPT) polarization transfer block followed by a 90° BIR4 excitation pulse. Parameters were TR = 2s, sweep width = 10 000 Hz, number of points = 4096. B, Dynamic ^1^H images of hyperpolarized [^15^N_2_]urea. The images were acquired with a 2D EPI sequence after successive polarization transfer blocks. Imaging parameters were FOV = 32 × 32 × 1 mm, TR = 1s, bandwidth = 250 kHz, matrix size = 32 × 32. The [^15^N_2_]urea concentration was 50 mM in both cases

Partial transfer of polarization from ^15^N to urea protons in [^15^N_2_]urea using IRRUPT can be used for dynamic spectral acquisition (Figure 5A) or for imaging (Figure 5B). The 90 Hz splitting in the urea ^1^H spectra is due to the ^1^H‐^15^N coupling.^28^ The remaining signal at the end of the dynamic spectral acquisition (Figure 5A) is residual signal from water protons.

### 3.4 Comparison of direct and indirect detection of [^15^N_2_]urea in interleaved acquisitions

**FIGURE 6:**
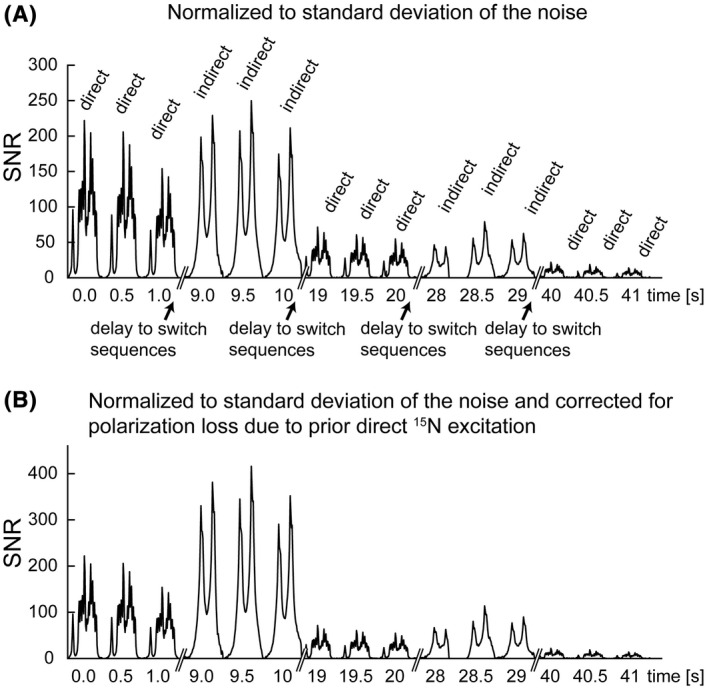
Interleaved direct ^15^N detection and indirect ^1^H detection of hyperpolarized [^15^N_2_] urea. A, The spectra were scaled so that the noise was the same in all the spectra. B, Indirect detection spectra additionally corrected for depletion of polarization due to the prior direct detection experiment. SNR, signal‐to‐noise ratio

The SNR in the indirect detection spectra recorded immediately after the direct detection spectra was significantly higher (Figure 6A). There was an ~8 s delay in changing from one pulse sequence to the next. After correcting for depletion of polarization in the preceding direct detection experiment there was a 2.09 ± 0.31(SD)‐fold improvement in SNR in the indirect detection experiment (Figure 6B). Each indirect measurement led to 20% depletion of the ^15^N polarization. Comparing spectrum 1 with 2 and 2 with 3, and similarly spectrum 7 with 8 and 8 with 9 shows that each of the direct acquisitions depleted 13 ± 7 (SD)% of the polarization, which corresponds to a FA of 30° (acos(0.87) = 30°). To compare the SNR of the directly and indirectly detected spectra, we corrected the SNR improvement factor of 2.09 ± 0.31 by sin30°/sin37°, which corrects for the fact that the indirect experiment depleted 20% of the ^15^N polarization (cos(37°) = 0.8) whereas the direct detection experiment depleted 13% of the polarization. This gives a corrected improvement in the SNR of 1.72 ± 0.25. The improvement in SNR was less than expected, reflecting a poorer than expected performance of the ^1^H circuit in the dual‐tuned ^1^H/^15^N transmit/receive surface coil. The ratio of the SNRs in ^1^H and ^15^N spectra acquired using this coil from thermally polarized 4M [^15^N_2_]urea was 59.9. When corrected for the number of contributing nuclei per molecule (four protons, two ^15^N nuclei) and the different thermal polarizations calculated using Equation (1) this gave an effective SNR enhancement (*ε*) of 6.07 when detecting ^1^H versus ^15^N for a given level of polarization. For example, if the SNR of an ^15^N acquisition is 10, the SNR of a ^1^H acquisition at the same nucleus concentration and polarization will be 60.7. This value for ε was less than an expected value of 54.92, if coil noise dominates, and a value of 9.86 if sample noise dominates (Equation 10).

### 3.5 Spontaneous transfer of polarization between ^15^N and ^1^H in hyperpolarized [^15^N_2_]urea

**FIGURE 7:**
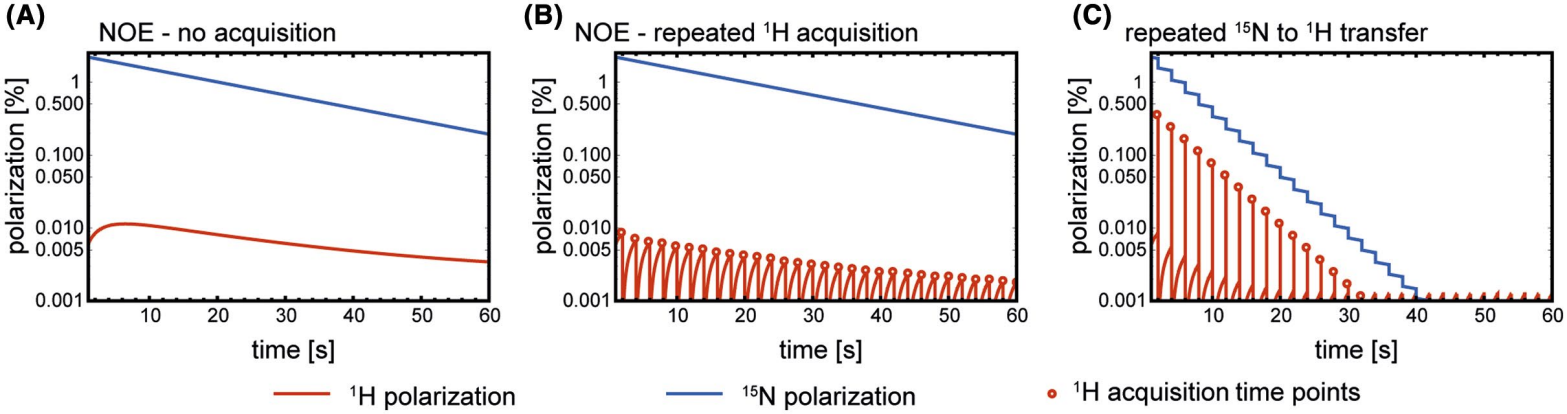
Simulation of polarization transfer due to cross relaxation between ^15^N and ^1^H in hyperpolarized [^15^N_2_]urea, where ^15^N hyperpolarization at *t = *0 is 2.3%. A, Time dependence of the ^15^N and ^1^H polarizations. B, ^15^N and ^1^H polarizations following a series of 90° ^1^H pulses applied at 2 s intervals. C, ^15^N and ^1^H polarizations following repeated application of the ImpeRfection RobUst Partial Transfer (IRRUPT) pulse sequence at 2 s intervals. NOE, nuclear Overhauser enhancement

Several studies have reported spontaneous transfer of hyperpolarization from a low γ nucleus to protons via cross relaxation.^29^, ^30^, ^31^, ^32^, ^33^ Using the measured cross relaxation rates (Figure 4B) we simulated transfer of polarization via cross relaxation and compared it with that obtained via J‐coupling using the IRRUPT pulse sequence. Polarization transfer via the spin coupling between ^15^N and ^1^H gave up to a 50‐fold higher proton polarization than that obtained via cross relaxation, although inevitably, because it depleted the ^15^N polarization more rapidly, this was sustained over a shorter period of time (Figure 7).

## 4 DISCUSSION

The signal detected by a receiver coil increases with the square of the detection frequency, ν^2^. However, coil and sample noise also increase as ν^1/4^ to ν, depending on the source of the noise. Therefore, the overall increase in the SNR as a function of ν is given by Wang et al^11^ and Hoult and Lauterbur^34^:

(10) $$\begin{array}{c} SNR\propto \frac{\nu^{2}}{{\left({\alpha a}^{2}\nu^{1/2}+{\beta \nu }^{2}b^{5}\right)}^{1/2}} \end{array}$$

where a and b are coil geometry parameters and α and β are weightings for the two sources of noise, where α represents coil noise and β sample noise. Equation 10 can be used when comparing the SNR of a direct detection experiment with a 90° pulse with an indirect detection experiment in which all the available polarization is transferred with perfect efficiency to the coupled high γ nucleus. However, for dynamic measurements only small portions of the hyperpolarization should be used at any one time, either by using a small FA pulse in the direct detection experiment or by using partial polarization transfer in the indirect experiment. In this situation we also need to consider the residual polarization left following each excitation because this represents the longitudinal magnetization available for subsequent excitation.^35^ The detectable transverse magnetization in a direct detection experiment depends on the FA thus:

(11) $$\begin{array}{c} {\left(M_{\mathrm{xy}}\right)}_{direct\;detect}\;\propto \;\sin FA \end{array}$$

Expressing the detectable magnetization in terms of the z‐magnetization used $\left({\left(M_{z}^{\mathrm{hyp}}\right)}_{\mathrm{used}}\right)$ then:

(12) $$\begin{array}{c} {\left(M_{\mathrm{xy}}\right)}_{direct\;detect}\propto \sqrt{1-{\left[1-{\left(M_{z}^{\mathrm{hyp}}\right)}_{\mathrm{used}}\right]}^{2}} \end{array}$$

In an indirect detection experiment the detected transverse magnetization in the higher γ nucleus is proportional to the gain from the higher precession frequency (*γ*_1H_/*γ*_15N_) multiplied by the z‐magnetization used ${\left(M_{z}^{\mathrm{hyp}}\right)}_{\mathrm{used}}$ in the lower γ nucleus:

(13) $$\begin{array}{c} {\left(M_{\mathrm{xy}}\right)}_{indirect\;detect}\propto \frac{\gamma_{\mathrm{high}}}{\gamma_{\mathrm{low}}}{\left(M_{z}^{\mathrm{hyp}}\right)}_{\mathrm{used}} \end{array}$$

**FIGURE 8:**
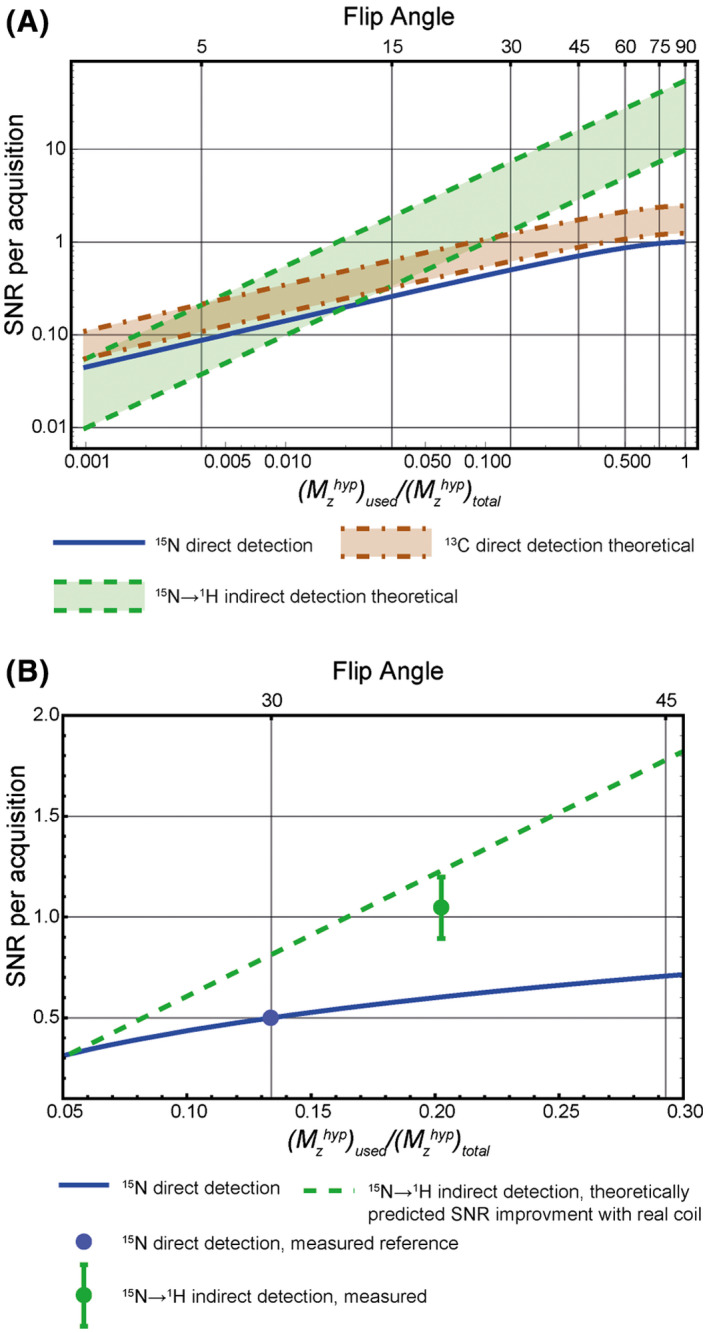
A, Theoretical signal‐to‐noise ratio (SNR) improvements for indirect detection as a function of flip angle and magnetization used, assuming perfect detection coils and equal levels of polarization for ^15^N and ^13^C, where the number of ^15^N spins is double the number of ^13^C spins, as is the case when comparing [^15^N_2_]urea with [1‐^13^C]urea. The upper and lower bounds for the SNR improvement were calculated for the situation when either coil noise (upper bound) or sample noise (lower bound) solely determined ε (Equation 10). B, Calculated and measured SNR improvement for indirect detection as a function of flip angle and magnetization used. The dashed green line is the theoretical SNR improvement with the dual tuned coil used here and the experimentally determined value for ε of 6.07

Comparing Equations (12) and (13) shows, that if

(14) $$\begin{array}{c} \frac{\gamma_{\mathrm{high}}}{\gamma_{\mathrm{low}}}{\left(M_{z}^{\mathrm{hyp}}\right)}_{\mathrm{used}}>\sqrt{1-{\left[1-{\left(M_{z}^{\mathrm{hyp}}\right)}_{\mathrm{used}}\right]}^{2}} \end{array}$$

then transferring polarization to the higher γ nucleus will result in greater transverse magnetization than would be obtained by direct detection of the lower γ nucleus. However, the actual gain in SNR will depend on coil performance at the two different resonance frequencies. For indirect detection versus direct detection in a polarization transfer experiment with perfect transfer efficiency, which is effectively the case here (see Figure 3C,F), then the gain in SNR is given by:

(15) $$\begin{array}{c} \frac{SNR_{15N\to 1H}}{SNR_{15N}}=\frac{\varepsilon {\left(M_{z}^{\mathrm{hyp}}\right)}_{\mathrm{used}}}{\sqrt{1-{\left[1-{\left(M_{z}^{\mathrm{hyp}}\right)}_{\mathrm{used}}\right]}^{2}}} \end{array}$$

where ε is the coil performance given by Equation (10). This is illustrated in Figure 8A, where the SNR of direct ^15^N detection with a 90° pulse is assumed to be 1. The SNR improvement for different detection strategies (^15^N direct detection, ^15^N → ^1^H indirect detection, or ^13^C direct detection) as a function of the magnetization used per acquisition are indicated. The upper and lower bounds for the improvement in SNR are determined by whether coil or sample noise dominates respectively. Direct experimental measurements of ε gave a value of 6.07, which was less than the lower bound given by Equation (10), reflecting poorer than expected performance of the ^1^H circuit in the dual tuned coil. However, with this experimentally determined value for ε there was good agreement between the theoretical gain in SNR for indirect ^15^N detection and that measured experimentally (Figure 8B).

Indirect detection of hyperpolarized [^15^N_2_]urea is an advantage over direct detection of [^15^N_2_]urea if more than 2% of the ^15^N polarization is used in each transfer, and over direct detection of [^13^C]urea if more than 11% of the ^15^N polarization is transferred at each step (Figure 8A), assuming that sample noise dominates and ε is only 9.86. With better coil performance these percentages are decreased such that if coil noise dominates, ie, the gain from detecting ^15^N polarization via spin‐coupled protons is fully realized, then indirect ^15^N detection is always an improvement over direct detection and an improvement over direct detection of [^13^C]urea if only 0.4% of the ^15^N polarization is transferred to proton. However, in the comparison with detection of [^13^C]urea there are other factors to consider. Although the [^15^N_2_]urea T_1_ is over 200 s in ^2^H_2_O, which allows the hyperpolarized material to be stored prior to injection, the T_1_ in water measured here was only 24 s and values as low as 9.8–12.9 s have been measured in blood,^2^ whereas we have measured a T_1_ for [^13^C]urea at 7 T in vivo of ~16 s^36^ and values of 43 s (at 11.7 T)^37^ and 78 s (at 3 T)^38^ have been measured in solution. Therefore, the polarization of [^13^C]urea will persist for longer in vivo than that for [^15^N_2_]urea, allowing dynamic imaging over longer time frames. The other problem with [^15^N]urea is that the achievable polarization level has so far been lower than for [^13^C]urea. Here we achieved polarizations of 6.2% for [^13^C]urea vs 2.3% for [^15^N_2_]urea, which are similar to levels reported previously,^2^, ^17^, ^18^ although values of 37% ^13^C polarization and 7.8% ^15^N polarization have also been reported.^39^ Note that indirect detection of [^13^C]urea is not feasible because the coupling constant with the protons, which are in exchange with solvent protons where the exchange rate is fast compared to the coupling constant, is weak and not observed. The sequence described here could also be used to detect hyperpolarized ^15^N via indirectly bonded protons, for example α‐trideuteromethylglutamine via the C2 proton, where in the absence of directly bonded protons the ^15^N T_1_ is much longer, although the J‐coupling will be weaker and therefore the efficiency of polarization transfer reduced. This glutamine derivative has been suggested as an alternative to [^13^C]urea for imaging tissue perfusion.^3^ The ^15^N in this molecule can be polarized to ~10% and in the rat kidney in vivo has a T_1_ of 146 s as compared to 18 s for [^13^C]urea.

Although polarization transfer sequences based on J‐coupling have been used previously with hyperpolarized ^13^C‐labeled substrates to produce hyperpolarized proton spectra^6^, ^7^, ^8^, ^9^ and images^10^, ^11^ and with hyperpolarized ^15^N labelled substrates to produce spectra,^12^, ^13^, ^14^ this is the first experimental demonstration of partial transfer, which with urea would be required for dynamic imaging of tissue perfusion. Simulations using measured cross relaxation rates showed polarization transfer via spin coupling can give orders of magnitude higher proton polarization than that obtained via cross relaxation^26^, ^29^, ^31^, ^32^, ^33^ (Figure 7). Moreover, the degree of transfer can be controlled to balance proton signal enhancement with the duration of the ^15^N hyperpolarization.

To summarize, we have shown that partial polarization transfer from ^15^N to ^1^H using a modified BINEPT sequence can be used for dynamic imaging of hyperpolarized [^15^N_2_]urea. If more than 10% of the ^15^N polarization is used in each acquisition then the sequence can give better sensitivity than direct ^13^C detection of [^13^C]urea for similar levels of hyperpolarization, and with full transfer of polarization can give an SNR that is 8.0‐22.4‐fold greater, depending on coil performance and whether coil or sample noise dominates. However, although we have used adiabatic pulses, implementation of this sequence in vivo will likely require the much better B_1_ field of a volume transmit coil, as was required for ^1^H detection of hyperpolarized [1‐^13^C]lactate in vivo.^10^, ^11^

## CONFLICT OF INTEREST

KMB holds patents with GE Healthcare on some aspects of DNP technology.

## Supporting information


**TEXT S1** Calculating the outcome of the ImpeRfection RobUst Partial Transfer (IRRUPT) sequence
