# Supplementary material for: Increasing the sensitivity of hyperpolarized [15N2]urea detection by serial transfer of polarization to spin‐coupled protons
Source: Magn Reson Med. 2020 Mar 10;84(4):1844–56. doi: 10.1002/mrm.28241 (PMC8629126; doi:10.1002/mrm.28241)
Supplement: Supplementary file 1 — TEXT S1 Calculating the outcome of the ImpeRfection RobUst Partial Transfer (IRRUPT) sequence [file MRM-84-1844-s001.docx]

Supporting information

Increasing the sensitivity of hyperpolarized [^15^N_2_]urea detection by serial transfer of polarization to spin-coupled protons

Felix Kreis^1^, Alan J. Wright^1^, Vencel Somai^1^, Rachel Katz-Brull^2^, Kevin M. Brindle^1,3^.

^1^Cancer Research UK Cambridge Institute, University of Cambridge, Cambridge, CB2 0RE, United Kingdom

^2^Department of Radiology, Hadassah Medical Center, Faculty of Medicine, Hebrew University of Jerusalem, Jerusalem

91120, Israel

^3^Department of Biochemistry, University of Cambridge, Cambridge, CB2 1GA, United Kingdom

### Calculating the outcome of the ImpeRfection RobUst Partial Transfer (IRRUPT) sequence

The density operator $\hat{\rho}$ is calculated step-by-step ($\hat{\rho}_{1\ldots9}$) in a composite pulse sequence analog of the IRRUPT pulse sequence, whereby the phase term, $\delta$ , splits transferred polarization between the I and S spins:

**S**: [90°_y_-𝜏_1_-180°_(y+π+δ/2)_-𝜏_2_-90°_y_]-𝜏*_3_*-[90°_y_180°_(y+ 3π/2)_90°_y_]-𝜏*_4_*-

**I**: [90°_y_-𝜏_1_-180°_(y+π)_-𝜏_2_-90°_y_]-𝜏*_3_*-[90°_y_180°_(y+3π/2)_90°_y_]- 𝜏*_4_*- Acq

A two spin IS system with the Hamiltonian $\hat{H}=2\pi J\hat{I}_{z}\hat{S}_{z}$ was assumed for illustration. The calculations can be generalized to any I_N_S system. We define the delays 𝜏 =2𝜏*_1_ =2*𝜏*_2_*, 𝜏*_3_ =*𝜏*_4_=1/(4J)* and the polarization levels $\mathbb{B}_{I}$ (hyperpolarized spins) and $\mathbb{B}_{S}$ (detection spins). The initial operator is:

$$\hat{\rho}_{1}=\mathbb{B}_{I}\hat{I}_{z}+\mathbb{B}_{S}\hat{S}_{z}$$

The polarization in the nuclei receiving the polarization is assumed to be zero ($\mathbb{B}_{I}=0)$ before the beginning of the pulse sequence, leading to

$$\hat{\rho}_{1}=\mathbb{B}_{S}\hat{S}_{z}$$

$$\downarrow\left( \frac{\pi}{2} \right)_{y}^{I}+\left( \frac{\pi}{2} \right)_{y}^{S}$$

$$\hat{\rho}_{2}=\mathbb{B}_{S}\hat{S}_{x}$$

$$\downarrow\pi J\tau/2$$

$$\hat{\rho}_{3}=\mathbb{B}_{S}\cos\left( \pi J\frac{\tau}{2} \right)\hat{S}_{x}+2\mathbb{B}_{S}\sin\left( \pi J\frac{\tau}{2} \right)\hat{S}_{y}\hat{I}_{z}$$

$$\downarrow\left( \pi\right)_{y+\pi}^{I}+\left( \pi\right)_{y+\pi+\delta/2}^{S}$$

$$\hat{\rho}_{4}=-\mathbb{B}_{S}\cos\left( \delta\right)\cos\left( \pi J\frac{\tau}{2} \right)\hat{S}_{x}+\mathbb{B}_{S}\sin\left( \delta\right)\cos\left( \pi J\frac{\tau}{2} \right)\hat{S}_{y}-2\mathbb{B}_{S}\sin\left( \delta\right)\sin\left( \pi J\frac{\tau}{2} \right)\hat{S}_{x}\hat{I}_{z}-2\mathbb{B}_{S}\cos\left( \delta\right)\sin\left( \pi J\frac{\tau}{2} \right)\hat{S}_{y}\hat{I}_{z}$$

$$\downarrow\pi J\tau/2$$

$$\hat{\rho}_{5}=-\mathbb{B}_{S}\cos\left( \delta\right)\cos\left( \pi J\tau\right)\hat{S}_{x}+\mathbb{B}_{S}\sin\left( \delta\right)\cos\left( \pi J\tau\right)\hat{S}_{y}-2\mathbb{B}_{S}\sin\left( \delta\right)\sin\left( \pi J\tau\right)\hat{S}_{x}\hat{I}_{z}-2\mathbb{B}_{S}\cos\left( \delta\right)\sin\left( \pi J\tau\right)\hat{S}_{y}\hat{I}_{z}$$

$$\downarrow\left( \frac{\pi}{2} \right)_{y}^{I}+\left( \frac{\pi}{2} \right)_{y}^{S}$$

$$\hat{\rho}_{6}=\mathbb{B}_{S}\cos\left( \delta\right)\cos\left( \pi J\tau\right)\hat{S}_{z}+\mathbb{B}_{S}\sin\left( \delta\right)\cos\left( \pi J\tau\right)\hat{S}_{y}+2\mathbb{B}_{S}\sin\left( \delta\right)\sin\left( \pi J\tau\right)\hat{I}_{x}\hat{S}_{z}-2\mathbb{B}_{S}\cos\left( \delta\right)\sin\left( \pi J\tau\right)\hat{S}_{y}\hat{I}_{x}$$

From here we use the following abbreviations:

$$\iota=\cos\left( \delta\right)\cos\left( \pi J\tau\right)$$

$$\kappa=\sin\left( \delta\right)\cos\left( \pi J\tau\right)$$

$$\lambda=\sin\left( \delta\right)\sin\left( \pi J\tau\right)$$

$$\mu=\cos\left( \delta\right)\sin\left( \pi J\tau\right)$$

The next step is then:

$$\downarrow\pi J 1/(4 J)$$

$$\hat{\rho}_{7}=\mathbb{B}_{S}\iota\hat{S}_{z}+\mathbb{B}_{S}\kappa\frac{1}{\sqrt{2}}\hat{S}_{y}-2\mathbb{B}_{S}\kappa\frac{1}{\sqrt{2}}\hat{S}_{x}\hat{I}_{z}+2\mathbb{B}_{S}\lambda\frac{1}{\sqrt{2}}\hat{I}_{x}\hat{S}_{z}+\mathbb{B}_{S}\lambda\frac{1}{\sqrt{2}}\hat{I}_{y}-2\mathbb{B}_{S}\mu\hat{S}_{y}\hat{I}_{x}$$

$$\downarrow\left( \frac{\pi}{2} \right)_{y}^{I}+\left( \frac{\pi}{2} \right)_{y}^{S}$$

$$\downarrow\left( \pi\right)_{y+3\pi/2}^{I}+\left( \pi\right)_{y+3\pi/2}^{S}$$

$$\downarrow\left( \frac{\pi}{2} \right)_{y}^{I}+\left( \frac{\pi}{2} \right)_{y}^{S}$$

$$\hat{\rho}_{8}=-\mathbb{B}_{S}\iota\hat{S}_{z}-\mathbb{B}_{S}\kappa\frac{1}{\sqrt{2}}\hat{S}_{y}+2\mathbb{B}_{S}\kappa\frac{1}{\sqrt{2}}\hat{S}_{x}\hat{I}_{z}-2\mathbb{B}_{S}\lambda\frac{1}{\sqrt{2}}\hat{I}_{x}\hat{S}_{z}-\mathbb{B}_{S}\lambda\frac{1}{\sqrt{2}}\hat{I}_{y}+2\mathbb{B}_{S}\mu\hat{S}_{y}\hat{I}_{x}$$

$$\downarrow\pi J 1/(4 J)$$

$$\hat{\rho}_{9}=-\mathbb{B}_{S}\iota\hat{S}_{z}+2\mathbb{B}_{S}\kappa\hat{S}_{x}\hat{I}_{z}-\mathbb{B}_{S}\lambda\hat{I}_{y}+2\mathbb{B}_{S}\mu\hat{S}_{y}\hat{I}_{x}$$

Resolving ι, κ, λ and μ gives:

$$\hat{\rho}_{9}=-\mathbb{B}_{S}\cos\left( \delta\right)\cos\left( \pi J\tau\right)\hat{S}_{z}+2\mathbb{B}_{S}\sin\left( \delta\right)\cos\left( \pi J\tau\right)\hat{S}_{x}\hat{I}_{z}-\mathbb{B}_{S}\sin\left( \delta\right)\sin\left( \pi J\tau\right)\hat{I}_{y}-2\mathbb{B}_{S}\cos\left( \delta\right)\sin\left( \pi J\tau\right)\hat{S}_{y}\hat{I}_{x}$$

Hence, the remaining hyperpolarization on the S spins is described by $-B_{S} \cos\left( \delta\right)\cos\left( \pi J\tau\right)$ while the transferred polarization on the I spins is described by $-B_{S} \sin\left( \delta\right)\sin\left( \pi J\tau\right)$ . For I_N_S spin systems the remaining hyperpolarization on the S spins can be described by $-\mathbb{B}_{S} \cos\left( \delta\right){\cos\left( \pi J\tau\right)}^{N}$ while the sum of the transferred polarization on the I spins in the higher order spin system is described by $-\mathbb{B}_{S} N\sin\left( \delta\right)\sin\left( \pi J\tau\right)$ . This shows that changing $\delta$ and $\tau$ varies the amount of transferred polarization. Optimal values for $\delta$ and $\tau$ that lead to polarization transfer are similar to those discussed by Norton and Weitekamp (1). This composite pulse sequence analog is equivalent to the IRRUPT pulse sequence with adiabatic pulses in terms of net rotations, but the paths taken by the magnetization vectors differ. In this sense it follows the arguments of Merkle et al. (2) where the BINEPT sequence is explained using a composite pulse sequence analog.

**References**

1. Norton VA, Weitekamp DP. Communication: Partial polarization transfer for single-scan spectroscopy and imaging. J. Chem. Phys. 2011;135 doi: 10.1063/1.3652965.

2. Merkle H, Wei H, Garwood M, Uǧurbil K. B1-insensitive heteronuclear adiabatic polarization transfer for signal enhancement. J. Magn. Reson. 1992;99:480–494 doi: 10.1016/0022-2364(92)90204-K.
